# Patients as healthcare consumers in the public and private sectors: a qualitative study of acupuncture in the UK

**DOI:** 10.1186/1472-6963-11-129

**Published:** 2011-05-27

**Authors:** Felicity L Bishop, Fiona Barlow, Beverly Coghlan, Philippa Lee, George T Lewith

**Affiliations:** 1 School of Medicine, University of Southampton, Aldermoor Health Centre, Aldermoor Close, Southampton SO16 5ST, UK; 2 Health Experiences Research Group, University of Oxford Department of Primary Health Care, Rosemary Rue Building, Old Road Campus, Headington, Oxford OX3 7LF, UK; 3 School of Psychology, University of Surrey, Guildford, GU2 7XH, UK

## Abstract

**Background:**

The aim of this study was to compare patients' experiences of public and private sector healthcare, using acupuncture as an example. In the UK, acupuncture is popular with patients, is recommended in official guidelines for low back pain, and is available in both the private sector and the public sector (NHS). Consumerism was used as a theoretical framework to explore patients' experiences.

**Methods:**

Semi-structured face-to-face interviews were conducted in 2007-8 with a purposive sample of 27 patients who had recently used acupuncture for painful conditions in the private sector and/or in the NHS. Inductive thematic analysis was used to develop themes that summarised the bulk of the data and provided insights into consumerism in NHS- and private practice-based acupuncture.

**Results:**

Five main themes were identified: value for money and willingness to pay; free and fair access; individualised holistic care: feeling cared for; consequences of choice: empowerment and vulnerability; and "just added extras": physical environment. Patients who had received acupuncture in the private sector constructed detailed accounts of the benefits of private care. Patients who had not received acupuncture in the private sector expected minimal differences from NHS care, and those differences were seen as not integral to treatment. The private sector facilitated consumerist behaviour to a greater extent than did the NHS, but private consumers appeared to base their decisions on unreliable and incomplete information.

**Conclusions:**

Patients used and experienced acupuncture differently in the NHS compared to the private sector. Eight different faces of consumerist behaviour were identified, but six were dominant: consumer as chooser, consumer as pragmatist, consumer as patient, consumer as earnest explorer, consumer as victim, and consumer as citizen. The decision to use acupuncture in either the private sector or the NHS was rarely well-informed: NHS and private patients both had misconceptions about acupuncture in the other sector. Future research should evaluate whether the differences we identified in patients' experiences across private and public healthcare are common, whether they translate into significant differences in clinical outcomes, and whether similar faces of consumerism characterise patients' experiences of other interventions in the private and public sectors.

## Background

Patients in the UK typically access health care through the National Health Service (NHS), which is free to all at the point of use. They also have the option to access health care through the private sector, which is paid for through private insurance schemes or directly out-of-pocket. The NHS is dominant: approximately 85% of healthcare funding is from the state, and the private sector is used by 10-22% of the population [1,2]. According to recent UK policy documents, this is likely to change and the private sector will probably have a greater role in the provision of UK health services in the future [3]. It is therefore timely to consider the impact of private and public provision on patients' access to and experiences of health services.

In the UK, much of the literature on healthcare utilisation across different sectors has focused on why patients use private sector healthcare. Large scale quantitative work suggests that seeking private sector healthcare (purchasing private insurance and/or receiving treatment) is more common among the middle-aged and affluent, and might also be associated with being male and more highly educated [4-8]. While personal financial resources are an important determinant of accessing private sector healthcare, psychological and sociological factors are also relevant. For example, those with more conservative political attitudes are more likely to use private healthcare and less likely to use public healthcare than others [2]. Perceived characteristics of public and private healthcare may also be relevant, and some have argued that people are pulled towards private healthcare more than they are pushed away from public healthcare [9]. Features that attract patients to private healthcare include the perceptions that senior doctors and better facilities are available [9], that care is easier to access and more patient-centred [8], that consultations and examinations are more thorough and waiting times are shorter [10]. Others have suggested that patients are pushed from public to private healthcare as a consequence of poor quality (specifically longer waiting lists) in the public sector [11].

Explanations of healthcare utilisation across sectors that are couched in terms of push and pull factors assume that patients conduct rational cost-benefit analyses and weigh up the pros and cons of using private and public sector healthcare before making an informed decision. This assumption is contentious and can be challenged. For example, an Australian study of people's reasons for having private medical insurance showed how 'public' reasons (waiting lists, choice of practitioner, quality of care, hotel services) were contradicted by people's actual personal experiences. These contradictions led the authors to argue that people do not engage in rational cost-benefit analyses when deciding to take private medical insurance, rather this decision is driven by trust in service providers [12]. Models of push and pull factors also offer limited insight into the finding that particular styles of therapeutic relationship seem to be more common in each sector; for example, in one study private patients experienced somewhat less paternalistic therapeutic relationships in primary care than did NHS patients [13]. A broader framework is thus needed that goes beyond a focus on the individual and can challenge the assumption that rational cost-benefit analyses underpin patients' decisions.

Consumerism offers a broad theoretical lens through which to consider how and why patients use healthcare in the private sector, and how this compares to healthcare utilisation in the public sector. With a focus on the individual, Gabriel and Lang [14] draw on multiple disciplines to provide an extensive typology that identifies 9 faces of the consumer (Table 1). This typology includes but is not limited to the conceptualisation of consumer as chooser, which is rather dominant in the policy-focused literature [15,16]. In particular, it also includes less appealing faces of consumerism (e.g. consumer as victim) which goes some way to taking into account the criticism that representations of patients as active, reflexive, autonomous consumers of healthcare inadequately reflect the embodied experience of illness and help-seeking [17]. With a focus on therapeutic relationships, Roter [18] describes consumerism as one of 3 prototypes of doctor-patient relationships (the other two being paternalism and mutuality). According to this framework, consumerism involves the doctor or therapist as a technical consultant obliged to provide the requested information and service(s), while the patient sets the agenda, makes decisions, and takes responsibility for those decisions. In paternalism, the doctor holds power as a benevolent expert obliged to act in the patient's best interests while controlling the agenda and making decisions, the patient has little or no voice. In mutuality, the doctor acts as advisor and both parties bring important resources and expertise to the process and jointly negotiate the agenda and decisions. Consumerism thus offers a framework for exploring patients' experiences of private and public healthcare that can be applied at the level of the individual patient as well as the therapeutic relationship.

**Table 1:** Gabriel and Lang's faces of consumerism [14] in the context of healthcare.

Face of consumerism	Description
Consumer as chooser	Makes an active and informed decision, selecting a healthcare option from a number of other options.

Consumer as communicator	Use of a form of healthcare conveys social and cultural meanings to others possibly involving social status.

Consumer as explorer	A particular form of healthcare is something new to be tried, amongst a universe of many possible new experiences.

Consumer as identity-seeker	The choice to use a particular form of healthcare contributes to the construction of a particular social identity.

Consumer as hedonist	Uses a particular form of healthcare to experience its positive emotional effects.

Consumer as victim	Is vulnerable and requires protection from unscrupulous healthcare practitioners or providers who might defraud them or otherwise cause harm.

Consumer as rebel	Uses a particular (unorthodox) form of healthcare as a form of resistance and rebellion against other (more mainstream) forms of healthcare.

Consumer as activist	Uses a particular form of healthcare as part of a broader political movement to challenge the status quo.

Consumer as citizen	Whose choice to use a particular form of healthcare is made within a community and thus has moral and social implications.

In the remainder of this paper, we use acupuncture as an example in which to examine the consumption of healthcare in the public and private sectors. Table 2 summarises the current state of acupuncture provision in the UK. Acupuncture provides a useful focus because it is popular with patients, particularly for pain-related conditions [19,20]. In addition, while acupuncture is predominantly used in the private sector it is also available in the NHS [19,21] and recent policy documents and clinical guidelines suggest its availability in the NHS may increase [22,23]. One study suggests that patients may experience acupuncture as holistic care differently according to whether they receive treatment in the NHS or private practice [24]. Finally, as a form of complementary and alternative medicine (CAM), acupuncture might facilitate consumerism in healthcare [17,25-28] and could thus provide a richer setting for a more nuanced exploration of consumerism than would be possible in a study of a mainstream biomedical modality. For example, Frank and Stollberg studied medical acupuncture in Germany and characterised patients as exhibiting passive rather than active consumerism: patients did evaluate the quality of their acupuncture, but neither sought information on nor compared different treatment options [29].

**Table 2:** Key facts about acupuncture in the UK

Practitioners
• Acupuncture can be delivered by acupuncturists as well as other practitioners who also offer acupuncture. • Major professional societies for acupuncturists include the British Acupuncture Council http://www.acupuncture.org.uk/, the Acupuncture Association of Chartered Physiotherapists http://www.aacp.org.uk, the British Medical Acupuncture Society http://www.medical-acupuncture.co.uk/.

Regulation
• Acupuncturists are not currently subject to statutory regulation in the UK. • Many UK acupuncturists are subject to statutory regulation in relation to their other professional identities (e.g. physiotherapist, biomedical doctor).

Education and Training
• Education and training in acupuncture is varied. Different styles (schools) of acupuncture are available in the UK. These include Western medical acupuncture [48] and Traditional Chinese acupuncture. • Private sector colleges provide degree courses in acupuncture. For example, the College of Integrated Chinese Medicine offers a 3 year course leading to a BSc (Hons) in Acupuncture http://www.acupuncturecollege.org.uk • Short training courses are available for some groups. For example, the British Medical Acupuncture Society provides a 4-5 day foundation course that confers a Certificate of Basic Competence for healthcare professionals who are regulated by statute in the UK.

Access
• Access to acupuncture in the private sector is typically via self-referral • Access to acupuncture in the NHS is typically via referral from a GP. Referrals might be either specifically for acupuncture (e.g. to an acupuncturist working in primary care) or more generally for secondary care (e.g. to a pain clinic or physiotherapy service in which acupuncture might then be delivered).

The aim of this study was to compare patients' experiences of public and private sector healthcare, using acupuncture as an example. In adopting the theoretical lens of consumerism, our objectives were 1) to explore the different ways in which patients in each sector might be considered to be individual healthcare consumers and 2) to explore the extent to which the conceptualisation of relationships as consumerist, paternalist, or mutualistic is helpful in understanding patients' experiences of acupuncture in the NHS and the private sector. We focus on acupuncture for pain because this is the most common reason for patients to seek acupuncture and much of the acupuncture provided on the NHS is for pain relief. A detailed analysis of acupuncture in these terms may reveal valuable insights that could relate to consumerism and the public versus private provision of other medical interventions.

## Methods

### Design

This qualitative study used semi-structured interviews and inductive thematic analysis. Each participant took part in a single interview with a researcher. This study was approved by the Southampton and South West Hampshire Research Ethics Committee (B) (07/HO504/196).

### Recruitment

We wanted to interview patients with recent experience (in the past 2 years) of using acupuncture for pain in either the NHS or private practice (or both). We only invited adult, English-speaking patients to take part in an interview. We aimed to recruit and interview participants until no new themes emerged around patients' beliefs about acupuncture provision in each healthcare sector. There is always the chance that the next person we could have interviewed might have prompted further thematic development; we thus balanced the diminishing insights to be gained from subsequent interviews against the resources required to conduct them. We sampled purposively to try to identify the range of beliefs that might be held about this topic, recruiting patients who had generally positive and generally negative experiences from private and NHS settings and some who had experienced acupuncture in both settings.

Participants were recruited from NHS and private acupuncture clinics in Hampshire, incorporating city, urban, and rural clinics. Practitioners were asked to identify any current patients meeting the inclusion criteria outlined above, and to give them a study invitation pack inviting them to take part in an interview about their experiences of acupuncture. Patients read the pack in their own time and contacted the researchers to arrange an interview. The study was also advertised within the University community and patient-led support and information networks such as Pain Concern to access people who had negative experiences of acupuncture.

As Table 3 shows, we successfully recruited 27 people spanning a wide range of ages and who had experienced acupuncture for a variety of conditions. Because many participants had experienced acupuncture on multiple separate occasions (sometimes years apart), and many did not know which style of acupuncture they had experienced, it was not possible to ascertain precisely how many had experienced different styles of acupuncture. However, at least one participant in each sector had received: Western medical acupuncture, Chinese or 5-element acupuncture, acupuncture from a Western medical doctor, and acupuncture from a physiotherapist. Participants had only received acupuncture from an osteopath or chiropractor in the private sector.

**Table 3:** Participants' Characteristics

Characteristic	Frequency (%)
Female	20 (74%)

Age (range)	23-75

Accessed acupuncture in:	

Private sector only	14 (52%)

Public sector only	5 (19%)

Both sectors	8 (30%)

Paid for private acupuncture*:	

With private medical insurance	4 (29%)

Out of pocket	8 (57%)

Both	2 (14%)

Primary Condition(s)	Chronic pain including pain in foot, knee, back, neck, shoulder.

*Information only available for "private sector only" participants. The financial cost of acupuncture in the private sector varies across individual practitioners, but clinics in the vicinity of this study typically charge around £50 for an initial appointment and £35 for a follow-up appointment.

### Data Collection

BC and PL conducted individual face-to-face semi-structured interviews in 2007-2008 with participants either in their home or at the University. The topic guide included open-ended questions about participants' experiences and thoughts before having acupuncture, their experiences of acupuncture treatments, and their reflections on the provision of acupuncture. If participants did not initiate these topics, the interviewer specifically asked about: reasons for use, consultations, outcomes, financial cost, clinic organisation and environment. Participants who had experiences of acupuncture within the NHS and private practice were asked to compare them. The interviews typically lasted approximately 60 minutes (range 35-105 minutes). With the participants' written informed consent, interviews were digitally recorded and transcribed verbatim; brief field notes were added to the transcripts. Participants were given a debriefing statement which thanked them for volunteering and reminded them of the aims of the study, they were offered a summary of the main themes discussed in their interview and they were invited to comment, if they wished, on the researchers' interpretation (no refutations of the researchers' interpretations were received).

### Data Analysis

Thematic analysis [30] was chosen as it is well-suited to the qualitative assessment of themes elaborated by many participants (it does not aim to produce in-depth interpretations of individuals' meanings). Qualitative analysis software was not used. At least three researchers discussed every analytic phase, helping to ensure that important issues were not overlooked and adding a diversity of viewpoints (FLB and GTL and BC/PL/FB). Following familiarisation with the data, inductive coding was used to encourage closeness to the data and to avoid premature links with existing theory. Labels were generated to summarise participants' talk. Splitting (creating more than one separate code from a single initial code) splicing (fusing smaller codes together) and linking (clustering smaller codes together around a common theme) were used to develop the coding and construct a representation of the data that remained true to the raw data (grounded) while moving towards higher order interpretation [30]. We met regularly to challenge each others' emerging interpretations and encourage reflexive awareness of any preconceptions we held about the topic. These meetings helped us to avoid producing an analysis that was idiosyncratic or selective. To enhance the rigour of our analysis, we deliberately sought contradictory evidence from the transcripts each time a theme was suggested. Working in this way we generated themes to describe and explain the bulk of the data. Table 4 illustrates the relationship between the final themes and initial lower level codes. Consistent with the inductive approach, the literature review was delayed until themes had been identified and elaborated. It was at this point that theories of consumerism were reviewed and related to our data. We also undertook an explicit search for data that did not fit with the emerging interpretation (deviant case analysis) to ensure openness to alternative interpretations and to test and define the limits of the emerging interpretation. In developing our analysis of this talk we paid particular attention to whether patients had experienced acupuncture on the NHS or the private sector or both, and have labelled the illustrative quotes below accordingly. Quotes were selected for typicality and eloquence in demonstrating themes.

**Table 4:** The relationship between themes and lower level codes

Theme	Examples of codes associated with theme
Value for money and willingness to pay	• Private acupuncture has to be something special • It is worth it if it works

Free and fair access	• Not fair that some people can't afford to pay for acupuncture • Acupuncture should be available to all (on the NHS)

Individualised holistic care: feeling cared for	• Treated as a person • NHS as a factory farm

Consequences of choice: empowerment and vulnerability	• Chance of getting 'ripped off' • Choice of (the best) practitioner • Patient has control over consultation agenda

"Just added extras": physical environment	• Expect more privacy in private sector • Private sector treatment essentially the same as NHS treatment

## Results

### Value for Money and Willingness to Pay

Participants who had accessed acupuncture on the private sector described being willing to pay for it because they valued their health. For some, acupuncture represented value for money; for others it was expensive but this was money well spent - provided, that is, the acupuncture worked. The converse was also true; if acupuncture was not working then it was not worth paying for. These participants were acting like the "consumer as chooser" [14] in that they were making an active decision to seek acupuncture. They particularly emphasised their willingness to pay for an effective treatment and judged their experiences on that basis. This suggests an additional face of consumerism in private sector healthcare: the consumer as pragmatist, who emphasises the likely health effects of their choices. Indeed, if acupuncture worked for them, then many of our private sector participants were not only willing to pay but were also willing to overcome additional practical barriers. For example, when NHS funding was withdrawn for John's acupuncture he paid to see the same practitioner privately despite his being located a considerable distance away. He did this because this practitioner's treatment was effective:

"Doesn't matter about the cost, you know. I used to have it on the [NHS] they paid for it for 2 years then they stopped paying, but I'd sooner pay the sixty five pounds and go all the way over to [acupuncturist] and have three quarters of an hour of excitement and come out tingling. I feel brilliant and I'm alright for at least three and a half weeks then." (John, both)

Private medical insurance was one means by which people could access acupuncture on the private sector without having to pay at point of use. Those participants who had private medical insurance did not talk about the cost of the insurance premiums. Instead, they talked about how their insurance made it possible for them to have acupuncture. Some participants who had accessed acupuncture in the private sector stopped treatment because they could not afford to continue, despite wanting more and feeling it could help. Lisa's choices in the private sector were limited by her financial situation: she did not have the resources to act on her preferred choice, to continue with an effective course of acupuncture.

"If it wasn't so prohibitively expensive I would use it a lot more but at thirty pounds plus a session every week [...] to do a really good course of acupuncture to get really good results I believe you should really give it a proper course, but I can't afford do use it like that... I stop as soon as I can simply because of the price." (Lisa, both)

While some participants were willing to pay for their health (to varying degrees), for others the private sector was simply unaffordable. They were unable to consume private sector healthcare even if they might have benefited from it. This disparity was acknowledged by private sector patients like Richard:

"I paid for it, fortunately I was in a position to be able to pay for it [...] I would feel sorry for people who couldn't." (Richard, private).

### Free and Fair Access

Not having to pay for acupuncture was an attractive feature of the NHS, for those who had accessed acupuncture on the NHS, in the private sector, and in both sectors. Unlike the private sector, the NHS was seen as supposedly offering equal, fair access to treatment for all. Here participants touched on more political themes, drawing on moral discourses to argue for NHS provision of acupuncture to facilitate access for people in pain who otherwise could not afford it and/or would not be aware of it. This concern with fairness suggests a "consumer as citizen" [14], with an interest in the social dimensions of the provision of healthcare: from this perspective, given that acupuncture is an effective pain management tool it should be offered freely to all who need it.

"I think it should be given as part of the NHS for it is a pain management tool [...] I know it's a very good pain management tool, it's unfair you have to go outside of the NHS and pay for it yourself [...] well maybe it was all in my head but it worked, and pain management is all about working isn't it? I mean you know, what works for one person's pain management doesn't matter, if you're living with chronic pain you've got to use what gets you by, don't you?" (Sue, private)

A lack of awareness of available services constituted a barrier to our participants accessing NHS acupuncture. Some participants who had only accessed acupuncture in the private sector were attracted to the idea of free acupuncture but did not know whether it was available on the NHS. Typically, participants in the private sector actively sought out acupuncture (although some received acupuncture after consulting a complementary therapist or physiotherapist practicing multiple modalities). They had researched acupuncture and made an active choice to use acupuncture rather than another therapy, but had not researched its availability in the NHS. Participants who chose acupuncture in the private sector were thus not making a fully informed choice (one of the characteristics of the "consumer as chooser"[14]), and might not be considered "smart consumers" [26].

"I wouldn't necessarily particularly want to pay out of pocket if I didn't have to, um, but I'm not aware how many there are in NHS" (Jane, private).

It was not just those participants who had used acupuncture in the private sector who were unaware of acupuncture services on the NHS. Often, those who had accessed acupuncture on the NHS had been pleasantly surprised to be offered it. Unlike those consuming acupuncture in the private sector, there was little evidence that participants using acupuncture on the NHS had made an active decision to seek out and try acupuncture. Instead, they typically accepted an offer of acupuncture made by a health care professional during the course of multi-modal treatment. Of the 8 participants who had experienced acupuncture in both sectors, only 1 had actively sought it in the NHS, while 7 had initiated their private sector acupuncture. Of the 5 participants who had only experienced acupuncture on the NHS, none had requested, suggested, or otherwise initiated acupuncture treatment. This passive acceptance of an offer of acupuncture is not characteristic of any of the faces of consumerism identified by Gabriel and Lang [14]. Instead, it resonates much more strongly with a paternalistic model of clinical interactions, where the expert benevolent practitioner determines the appropriate course of treatment (with the patient's consent).

"I just thought they were going to sort of give me exercises, medication, and that was about it really. I didn't think it was going to be alternatives. [...] So I thought it was quite good actually." (Tina, NHS).

Those participants who had used NHS acupuncture services did not necessarily continue to be passive recipients of acupuncture. Instead, they treated the offer of acupuncture as an opportunity to become an "explorer" [14], to try an unfamiliar treatment. This was positive for participants who had tried acupuncture in the NHS, discovered it worked for them, and were able subsequently to seek it out in the private sector. These participants reconfigured their experience of NHS acupuncture as a chance to "try before you buy", to experience and evaluate a therapy and so be in a position to make an informed decision about whether the financial cost of private sector acupuncture was going to be worth paying. From this perspective, the opportunistic and comparatively passive use of acupuncture in the NHS can be seen as a stepping stone towards a more informed, active, choice to consume acupuncture in the private sector.

"I am glad I am having it on the NHS really. I don't know if I'd have tried it if someone had said 'you've got to pay for it first'." (Michelle, both)

However, there was also a downside to the NHS providing a means of trying acupuncture. Those who believed themselves unable to afford the private sector were very concerned that they might discover an effective treatment that they would then be unable to access in the future.

### Individualised Holistic Care: Feeling Cared For

Participants valued the individualised, holistic care that they reported receiving in the private sector. They felt cared for, and linked this to the length and depth of consultations and to continuity of care. Practitioners were perceived as caring when they: allowed patients to communicate new health problems or concerns during treatment; took into account the patient's context, views, and desires; and provided treatments that were tailored to the individual patient. This is reminiscent of the mutuality model of doctor-patient relationships, in which an egalitarian partnership entails negotiation, respect, and joint decision-making.

"[private sector healthcare] is friendly, it's informal, it's not inhibitive, it's not institutional like a hospital. It enables you to consult with your medical practitioner on a range of topics that concern you, in other words when I'm on the couch as I said for acupuncture I can talk to him about the radiation colitis if I want to." (Paul, private)

Patients receiving acupuncture on the private sector typically contrasted positive descriptions of individualised care in the private sector with negative expectations of NHS treatments. They attributed individualisation not to acupuncture per se, but to acupuncture as delivered in the private sector. Len valued having more time to open up to his practitioner in the private sector than in the NHS; Jill felt that in the NHS she would be "shunted around from person to person" rather than seeing one acupuncturist; Gemma referred to the NHS as a "factory farm" and chose a private acupuncturist to get more individual attention.

Standardisation of treatment was seen as constraining individualisation and was raised as a disadvantage of NHS treatment by participants who both had and had not experienced it. NHS acupuncture was described as having a fixed number of treatments (too few) and a fixed duration of each treatment (too short). The standardisation of treatment in this way reduces opportunities for negotiation because many key decisions about treatment have already been made, probably encouraging paternalism rather than consumerism or mutuality. Participants understood standardisation as a consequence of the high volume of patients and the limited resources in the NHS - it was seen as a feature of the organisational structure, within which well-meaning individual practitioners had to work.

"I mean I loved going over there for it and they didn't want to stop it but they were trying to like carry it on, you know. But they just said there's no funding for it. They just done it out of a favour, really, I think, to try and help me get out of this pain." (Rebecca, NHS).

### Consequences of Choice: Empowerment and Vulnerability

Greater perceived choice and control were empowering features of private sector acupuncture. In particular, participants accessing acupuncture in the private sector valued: being able to start and stop a course treatment when they wanted to, rather than having to seek referral from a GP gatekeeper and/or join a waiting list before starting; being able to select their own practitioner whom they often described in glowing terms (and being confident that they would be treated by that practitioner); and participating in clinical decisions about, for example, the use of additional modalities and treatment frequency. In an extreme case, Peggy described her consumerist relationship with a practitioner to the extent that when she did not receive the treatment she requested, she switched practitioner. This is one of three examples in our data of a participant rejecting a practitioner through the mechanism of "exit", a key mechanism available to consumers dissatisfied with a service which cannot be returned in the same way as material goods [31].

"I went asking for acupuncture and he was sort of saying 'I don't think you should have acupuncture, I want you to try diet and I want to try other tests, perhaps you've got a sensitivity that's causing all this.' Well, ok, but it's going back to what I said about being in control of what I want to do." (Peggy, private)

In the NHS, the lack of choice available to individual patients had disempowering consequences. In particular, patients were disempowered by the paternalistic situation in which they neither knew what would happen after a first course of treatments, nor contributed to the decision to stop treatment.

"So I had six weeks of acupuncture which I felt did help a hell of a lot and it was a shame that I was only offered six weeks because, that's, I think when something is working, you know, where the patient should be able to say well look, that's working, why can't I go on with that?" (Len, both).

While the lack of voice was a problem in the NHS, too much choice in the private sector was associated with vulnerability and could thus be problematic. Some participants perceived a risk of being taken advantage of by unscrupulous practitioners, and were concerned about finding a suitable - trustworthy, qualified - acupuncturist in the private sector. Karen reported being "dubious" about the qualifications of acupuncturists based in shops, while Debra thought that being in pain exacerbated one's vulnerability when seeking treatment. This reflects the "consumer as victim" face of consumerism [14], reminds us of the embodied vulnerability and emotional dimensions of ill-health [17], and suggests that these participants did not feel sufficiently empowered or informed to judge the quality of a practitioner without some institutional protection or legitimisation.

### "Just Added Extras": Physical Environment

This final theme illustrates the disparity between participants' experiences and (somewhat stereotypical) expectations of private sector acupuncture. Participants who had only experienced NHS acupuncture typically expected only the physical environment to be different in the private sector. They believed that the essential features of acupuncture would be identical in each sector, as did some participants who had used acupuncture in both sectors.

"I don't know what else I would have if I was going private, I have no idea. Whether I would have the - you know, your own room, and your own...your bit of music and everything. But I mean the treatment would still be the same I guess. So all those are just added extras" (Michelle, NHS)

This suggests that consumers of private sector healthcare might be vulnerable to being seen by others as "hedonistic consumers" [14], using private sector acupuncture to benefit emotionally from a more pleasant physical environment. However, this face of consumption was not present in private sector participants' accounts. Indeed, patients who had only used acupuncture in the private sector rarely talked about the physical environment, and focused instead on the value for money and effectiveness of their treatment and their experiences of care, control, and choice as described above. Interestingly, the private sector did not always live up to its reputation for better hotel services: Ann's NHS acupuncture was in a "nicer room" than her private acupuncture.

## Discussion

Patients who had received acupuncture in the private sector constructed detailed accounts of the benefits of private care. They reported how, by going privately, they were availing themselves of greater choice and control and more holistic, personal, care. The value our participants placed on holistic and mutualistic therapeutic relationships echoes that reported in previous studies on acupuncture [32-35] but our participants attributed these features more to the private sector than to acupuncture per se. These psychosocial benefits featured more strongly than the physical environment of treatment, but the bottom line was that patients only continued to have and pay for acupuncture in the private sector if they felt it was working for them. Financial cost was the main barrier to accessing acupuncture in the private sector; others have similarly identified cost as deterring or curtailing private sector CAM use [36,37]. Patients who had not received acupuncture in the private sector expected better physical environments but otherwise no difference from NHS-based treatment. Similar findings have been reported for dentistry: people perceived greater access in the private sector but saw NHS dentistry as essentially the same as private sector dentistry [38]. Our participants constructed NHS acupuncture as a treatment that may have limited duration and/or scope but is otherwise of a similar (high) quality to private sector acupuncture. However, in the NHS, patients neither talked about having choice and control over their treatments, nor talked about acupuncture in holistic terms, although they did praise their individual practitioners. Others have noted the challenges of retaining a holistic approach to CAM in the NHS [39]. Overall, features of the private sector that were attractive to and valued by our patients echo those reported in other, conventional, healthcare settings, such as being able to choose a practitioner, greater perceived control over treatment, better quality care, and better 'hotel services' or physical environment [40-44].

Patients seeking acupuncture were seen to exhibit a variety of consumerist behaviours. Eight different faces of consumerism were identified, although the "smart consumer" was rare and the "consumer as hedonist" was only hinted at as an identity for others. These eight faces are summarised in Table 5. Of the nine faces of consumerism identified by Gabriel and Lang [14], the "consumer as chooser" resonates most closely with our findings. Choice has dominated conceptualisations of consumerism [15] and has a long history in UK health policy [45]. Our patients reported 'going private' in order to obtain the services, choice and control that they did not perceive were available to them in the NHS. They then negotiated their treatment in the private sector with varying degrees of commitment (or loyalty) to an individual practitioner and occasionally with the threat of exit to a new practitioner [31]. They emphasised the health effects of acupuncture, suggesting a "consumer as pragmatist". The "consumer as victim" was also identified and a reconfiguration of "consumer as explorer" to "consumer as earnest explorer" [25] might capture something about the patient who, in pain, is willing to try anything. The "consumer as citizen" was seen in patients seeking acupuncture in the NHS and in some of those in the private sector. At the level of the therapeutic relationship, consumerist relationships were rare even in the private sector, mutualistic relationships were described in both sectors, and elements of paternalism were seen particularly in the NHS, where they were facilitated by standardisation of treatment. Our NHS participants might be considered passive consumers in a similar way to the German patients who evaluated their experience of acupuncture but did not seek out information about treatment options [29]. Interestingly, when viewed in the context of subsequent healthcare seeking, the passive receipt of acupuncture in the NHS ("consumer as patient") could be reconfigured as a first exploratory step which facilitated a well-informed active decision to use acupuncture in the private sector.

**Table 5:** Faces of acupuncture consumerism identified in this study

Face of consumerism	Description
Consumer as smart [26] chooser	Makes an active and informed decision, selecting acupuncture from a number of other options and making an informed decision to access it in a specific setting. (Very rare in this study.)

Consumer as chooser*	Makes an active decision, selecting acupuncture from a number of other options.

Consumer as patient	An offer of acupuncture is accepted within the context of a therapeutic relationship.

Consumer as earnest explorer [25]*	Acupuncture is something new to be tried, amongst a universe of many possible therapies, in the context of an embodied need for treatment.

Consumer as pragmatist	Emphasises the likely effectiveness of acupuncture in a particular setting and the actual effectiveness of it once experienced.

Consumer as hedonist*	Uses a particular form of healthcare to experience its positive emotional effects (only seen as attributed to the private sector by NHS patients).

Consumer as victim [14]	Is vulnerable and requires protection from unscrupulous healthcare practitioners or providers who might defraud them or otherwise cause harm.

Consumer as citizen [14]	Whose choice to use private or public sector healthcare is made within the context of moral and social implications of private sector involvement in healthcare provision.

*Adapted from Gabriel and Lang [14]

Our findings are inconsistent with economic models that assume consumers make choices based on reliable, complete, and comprehensible information about their options. Similarly, the "smart consumerism" identified by Kelner and Wellman [26] was not prevalent among our participants. Kelner and Wellman's private-sector CAM users were informed about health issues and used lay referrals and personal judgment to make partly ideological and partly pragmatic choices to consult specific types of practitioners for specific health issues [26]. In our study, NHS and private patients both had misconceptions about acupuncture in the other sector. For example, NHS patients over-estimated the cost of private sector acupuncture and private patients under-estimated the availability of NHS acupuncture. This supports and extends previous studies which have demonstrated how patients' assumptions and beliefs about the nature of NHS and private services influence their decisions to seek private healthcare [41,46]. It provides further evidence of the importance of studying patients' beliefs, rather than modelling patients as undertaking 'rational' cost-benefit analyses and assuming that 'objective facts' about health services, such as official statistics about waiting lists, will determine patients' decision-making.

Some limitations of our study should be acknowledged. While our patients had received similar styles of acupuncture in each sector, and had received acupuncture from a similar range of practitioners, there may be differences in how acupuncture is practised across the sectors especially if we consider the NHS as a largely paternalistic institution dominated by orthodox medicine [47]: we will report separately a parallel study of acupuncturists. Most of our participants had experienced acupuncture in the private sector, and only 19% had only experienced acupuncture in the NHS. We suspect this probably reflects the availability of acupuncture in the different settings, but a greater number of participants who had only experienced NHS acupuncture might have enriched our understanding of that sector. Similarly, only a small minority of our participants were men and so there may be as yet unidentified ways in which men consume acupuncture in the NHS and/or private practice. It might have been helpful to interview participants more than once in order to trace their experiences over time, although we did successfully recruit some participants who had only experienced one episode of acupuncture treatment and others who had experienced multiple episodes over many years. We were only able to consider patients' accounts of relationships with acupuncturists: observational work is needed to examine the ways in which consumerist behaviour is evident in acupuncture consultations. We believe our findings are inevitably shaped by our interests, but that the credibility of our work is enhanced by our committed use of established research procedures and the range of perspectives we bring to this project: our backgrounds include psychology, sociology, biomedicine, and acupuncture; some of us have never received or practiced acupuncture, while GL has practiced acupuncture in both private and NHS clinics over the last 30 years; between us we hold a range of attitudes towards public and private healthcare and acupuncture.

## Conclusions

In conclusion, patients using acupuncture experienced it quite differently in the NHS compared to the private sector. They exhibited different forms and faces of consumerist behaviour, including consumer as chooser, consumer as pragmatist, consumer as patient, consumer as earnest explorer, consumer as victim, and consumer as citizen. Our findings may have relevance for treatments other than acupuncture, including other CAM therapies such as osteopathy which have a comparatively small presence in the NHS, and conventional therapies such as physiotherapy which are comparatively affordable in the private sector. Future research should evaluate whether the differences we identified in patients' experiences across private and public healthcare are common, whether they translate into significant differences in clinical outcomes, and whether similar faces of consumerism characterise patients' experiences of other interventions in the private and public sectors.

## List of Abbreviations

CAM: Complementary and alternative medicine; NHS: National Health Service; GP: General Practitioner;

## Competing interests

The authors declare that they have no competing interests.

## Authors' contributions

FLB conceived of and designed the study, supervised the data collection, participated in the analysis, and drafted the manuscript. FB contributed to the analysis. BC and PL carried out the interviews and participated in the analysis. GTL contributed to the design and analysis. All authors were involved in revising the manuscript and read and approved the final manuscript.

## Pre-publication history

The pre-publication history for this paper can be accessed here:

http://www.biomedcentral.com/1472-6963/11/129/prepub
